# Detection of Specific Antibodies against Toscana Virus among Blood Donors in Northeastern Italy and Correlation with Sand Fly Abundance in 2014

**DOI:** 10.3390/microorganisms8020145

**Published:** 2020-01-21

**Authors:** Silvia Morini, Mattia Calzolari, Giada Rossini, Nadia Pascarelli, Andrea Porcellini, Vanda Randi, Maria Carla Re, Alessandro Albieri, Paolo Bonilauri, Romeo Bellini, Nazli Ayhan, Remi Charrel, Stefania Varani

**Affiliations:** 1 Unit of Clinical Microbiology, Regional Reference Centre for Microbiological Emergencies (CRREM), St. Orsola-Malpighi University Hospital, 40138 Bologna, Italy; silvia-morini@libero.it (S.M.); giada.rossini@unibo.it (G.R.); 2 Laboratory of Entomology, Istituto Zooprofilattico Sperimentale della Lombardia e dell’Emilia Romagna “B. Ubertini” (IZLER), 42124 Reggio Emilia, Italy; mattia.calzolari@izsler.it (M.C.); paolo.bonilauri@izsler.it (P.B.); 3 Centro Regionale Sangue Emilia-Romagna, Maggiore Hospital, 40133 Bologna, Italy; nadia.pascarelli@ausl.bologna.it (N.P.); andrea.porcellini@ausl.bologna.it (A.P.); vanda.randi@ausl.bologna.it (V.R.); 4 Department of Experimental, Diagnostic and Specialty Medicine, University of Bologna, 40138 Bologna, Italy; mariacarla.re@unibo.it; 5 Department of Medical and Veterinary Entomology, Centro Agricoltura Ambiente ‘G. Nicoli’, 40014 Crevalcore, Italy; aalbieri@caa.it (A.A.); rbellini@caa.it (R.B.); 6 Unité des Virus Emergents (UVE: Aix Marseille Univ, IRD 190, INSERM 1207, IHU Méditerranée Infection), 13005 Marseille, France; nazliayhann@gmail.com (N.A.); remi.charrel@univ-amu.fr (R.C.)

**Keywords:** Toscana virus, viral meningitis, seroprevalence, arbovirus, sand fly, *Phlebotomus* spp., neutralization

## Abstract

Toscana virus (TOSV) is a Phlebovirus transmitted by phlebotomine sand flies and is an important etiological agent of summer meningitis in the Mediterranean basin. Since TOSV infection is often asymptomatic, we evaluated the seroprevalence in blood donors (BDs) in the Bologna and Ferrara provinces (Northeastern Italy)—the areas with the highest and lowest numbers of TOSV neuroinvasive cases in the region, respectively. A total of 1208 serum samples from BDs were collected in April–June 2014 and evaluated for the presence of specific TOSV-IgG by ELISA. The IgG-reactive samples were confirmed by indirect immunofluorescence assay (IIF) and by microneutralization test (MN). Serum samples were defined as positive for anti-TOSV IgG when reactive by ELISA and by at least one second-level test; TOSV seroprevalence was 6.8% in the Bologna province, while no circulation of TOSV was detected in the Ferrara province. Sand fly abundance in 2014 was also estimated by a geographic information system using a generalized linear model applied to a series of explanatory variables. TOSV seroprevalence rate was strongly associated with the sand fly abundance index in each municipality, pointing out the strong association between sand fly abundance and human exposure to TOSV.

## 1. Introduction

Toscana virus (TOSV) is an arthropod-borne virus belonging to the *Phlebovirus* genus of the *Phenuiviridae* family; this virus is transmitted by phlebotomine sand flies, mainly *Phlebotomus perfiliewi*, *Ph. perniciosus*, and *Ph. tobbi* in Europe [1,2,3]. TOSV was firstly isolated from *Ph. perniciosus* in Central Italy (Tuscany) in 1971 [4]; fifteen years later, TOSV infection was reported from returning travelers who had visited areas where the virus was initially described [5,6].

In the Mediterranean area, phleboviruses transmitted by sand flies belong to one of the following groups: (i) two International Committee on Taxonomy of Viruses (ICTV) recognized species, i.e., sandfly fever Naples (including TOSV and Naples virus) and Salehabad (including Salehabad and Arbia viruses) and (ii) two tentative species, i.e., sandfly fever Sicilian virus and Corfou virus [7]. Besides TOSV, the sandfly fever Sicilian (SFSV) and sandfly fever Naples (SFNV) viruses exhibit the widest geographical distribution within the Mediterranean region, which is likely related to the wide distribution of their proven vector (*Ph. papatasi*) in this area [4,8,9,10].

SFSV and SFNV are the etiologic agents of the “pappataci fever” characterized by fever, myalgia, and headache [11], while TOSV displays a strong neurotropism, being responsible for aseptic meningitis and meningoencephalitis, mainly associated with a favorable outcome [5,12]. Other phleboviruses have been related to human pathology; a novel phlebovirus that appeared genetically close to SFSV was detected during an outbreak of febrile syndrome among Greek soldiers stationed in Cyprus in 2002. From the location comes the provisional denomination of sandfly fever Cyprus virus (SFCV) [13,14].

In the past two decades, TOSV circulation has been increasingly reported in the Mediterranean basin; cases of neuroinvasive infection have been observed in residents of or travelers to Portugal, Spain, Italy, France, Cyprus, Turkey, and Greece [5,11]. Currently, TOSV is a prominent cause of summer meningitis and encephalitis in Mediterranean Europe [15].

In Italy, TOSV has been detected in several regions including Tuscany, Piedmont, Marche, Umbria, Lazio, Campania, and Sardinia [4]. Since 1999, autochthonous human cases of TOSV meningitis have been reported in the Emilia-Romagna region (Northeastern Italy), indicating local circulation of the virus [16]. In 2010, a surveillance plan to monitor human arboviral cases was adopted by the Emilia-Romagna region, leading to the detection of 61 cases of TOSV meningitis or meningoencephalitis in 2010–2012 [17] and 82 cases in summer–autumn 2013 [18]. Two sand fly species are present in the Emilia-Romagna region, i.e., *Ph. perfiliewi* and *Ph. perniciosus*, with an overwhelming presence of the first species [17].

Since TOSV infection is often asymptomatic [19], we hypothesized that the expected seroprevalence would be higher in areas with a higher number of TOSV cases. To verify this hypothesis, we performed a serosurvey for the identification of anti-TOSV antibodies among blood donors (BDs) in the Emilia-Romagna region. The study was carried out in April–July 2014, involving the central-eastern part of the region; sera were collected from BDs in areas with the highest and lowest number of neuroinvasive cases in previous years, i.e., the provinces of Bologna and Ferrara, respectively [17]. Serum samples were firstly evaluated for the presence of specific IgG by ELISA and only reactive samples were further tested by indirect immunofluorescence assay (IIF) and by microneutralization assay (MN). As a secondary aim, we explored the association between the observed TOSV seroprevalence and the estimated sand fly abundance in the study area.

## 2. Materials and Methods

### 2.1. Study Area and Sampling

We conducted a retrospective serological study in healthy Italian individuals volunteering for blood donation. In Italy, blood donation strictly follows laws and regulations from the Italian National Transfusion Network [20]. Blood samples were obtained from the Regional Blood Centre of Emilia-Romagna, Maggiore Hospital of Bologna (Italy), stored at 4 °C and sent to the Unit of Microbiology (St. Orsola-Malpighi University Hospital, Bologna, Italy) within 5 days of collection. Blood samples were centrifuged and stored at −80 °C. The study included BDs residing in the Bologna and Ferrara provinces, within the Emilia-Romagna region (4,446,220 inhabitants), Italy. From April to July 2014, 1008 serum samples from BDs residing in Bologna and 200 serum samples from BDs residing in Ferrara, respectively, were collected. The number of samples collected was related to the population’s density; Bologna province has around 1,000,000 inhabitants and Ferrara province around 300,000 inhabitants. The study was performed in accordance with the Declaration of Helsinki. Generic written informed consent for storage and future use for research purpose was obtained from each BD before sampling. Blood samples were coded and retrospectively analyzed by serological tests. As samples were anonymized, no further consent to participate to this study could be requested from blood donors, according to the Italian Data Protection Authority (Authorization n. 9/2016). All sera were stored at −80 °C until tests were performed.

### 2.2. Serological Tests for the Detection of Anti-TOSV IgG

**Figure 1:**
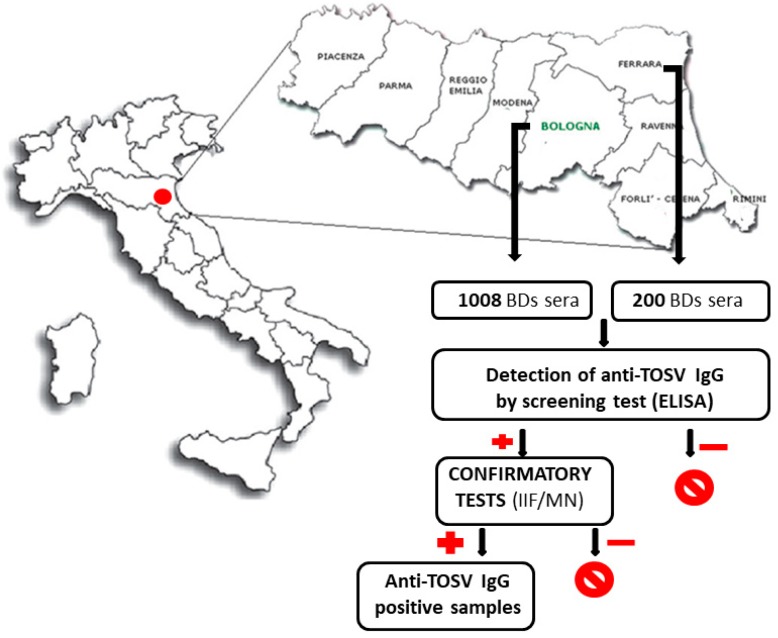
Work-flow of TOSV serosurvey. Blood donor (BD) sera were collected from the Bologna and Ferrara provinces (Northeastern Italy) and tested by ELISA for the presence of anti-TOSV IgG. Samples testing reactive by ELISA were confirmed by indirect immunofluorescence assay (IIF) and microneutralization test (MN).

Blood samples obtained from BD residing in Bologna (*N* = 1008) and Ferrara (*N* = 200) were firstly analyzed for the presence of antibody to TOSV by an immunoenzymatic assay and only positive sera were further tested by IIF and MN (Figure 1).

#### 2.2.1. Detection of Anti-TOSV IgG by ELISA

Each serum sample was first evaluated for the presence of specific TOSV-IgG using an in-house ELISA, as described [18]. Briefly, the viral antigen consisted of purified and inactivated virus, obtained from TOSV strain MRS2010-4319501 isolated in Marseille. The optical density (OD) values were normalized, and the ratio between normalized OD and the cut-off value was calculated. Samples presenting a ratio between normalized OD and a cut-off value higher than 1.1 were considered positive, while samples with a ratio between normalized OD and a cut-off value between 1.0 and 1.1 were considered borderline.

#### 2.2.2. IIF for Anti-TOSV IgG Detection

All positives and borderline ELISA samples were diluted 1:100 and tested with IIF for the detection of anti-TOSV IgG (Sandfly fever virus Mosaic 1, Euroimmun, Lubeck, Germany). IIF was performed following the manufacturer’s instructions. The assay simultaneously detects specific IgG against TOSV, SFNV, SFSV, and SFCV.

#### 2.2.3. Detection of Anti-TOSV and Anti-SFSV Neutralizing Antibody

MN was performed as described [18]. Briefly, 100 TCID50 (50% tissue culture infective dose) of TOSV (strain MRS2010-4319501) or SFSV (strain UVE/SFSV/1943/IT/Sabin, Ref#001v-EVA77 accessible at https://www.european-virus-archive.com/virus/sandfly-fever-sicilian-virus-strain-uvesfsv1943itsabin) were added to wells of a 96-well plate containing patient serum and incubated at 37 °C for 1 h. Subsequently, a 100 μL suspension of Vero cells containing approximately 2 × 10^5^ cells/mL was added to each well and incubated for 5 days. The microplates were then read under an inverted microscope, and the presence (neutralization titer at 10, 20, 40, 80 and 160) or absence (no neutralization) of cytopathic effect was noted.

### 2.3. Correlation between Sand Fly Abundance and TOSV Seroprevalence

#### 2.3.1. Estimation of Sand Fly Abundance

Sampling data obtained in the frame of the Emilia-Romagna regional surveillance plan for West Nile virus and leishmaniasis in 2014 were retrieved for modeling the abundance of sand flies. Data referred to the Bologna and Ferrara provinces; to improve the model also data from the neighboring Ravenna province were used. Data concerning 1720 sand flies, sampled between 3 July and 21 August 2014, were used; insects were collected in 29 out of the 62 monitored sites using attractive traps baited with carbon-dioxide overnight. The summary of entomological data utilized for the model implementation is reported in Table 1. A generalized linear model (GLM) applied to a series of remote sensing variables (vegetation index, bioclimatic indexes, land cover, elevation) was used to estimate the abundance of sand flies (sum of sand flies per site), which was the dependent variable in a Poisson regression. The first four variables ranked by the model were all linked to the temperature (the mean diurnal range, the isothermality, temperature seasonality, and the annual temperature range). The abundance model was carried out in R version 3.3.2 (R Development Core Team 2005) using the lme4, lattice, Tweedie, boot, raster, sp, and rgdal packages. The average of sand fly abundance was then estimated for each municipality through the obtained model (model details are reported in the Supplementary Materials).

#### 2.3.2. Data Analysis

A binomial logistic regression model was used to evaluate the correlation between the TOSV seroprevalence at each municipality and sand fly abundance (SA). To reduce the high variability typical of the vector population, the abundance was log10 transformed before binominal logistic regression model analysis was employed. For each municipality, results from the prevalence observed in BD (p+) were analyzed separately by means of binomial logistic regression with log10 SA as covariate.

Logistic (p+) = Ln [p+/(1 **−** p+)] = a + b (log10 SA) (1)

To avoid 0 prevalence results and to consider the uncertainty introduced by sampling on the true prevalence estimation, the mean of a beta function was used. Using Bayes’ theorem where no prior knowledge of the prevalence in the BD population is assumed, the fraction of positive donors could be assumed to follow a Beta distribution [21]; if we assume a uniform (0.1) prior distribution for p+ (the probability of being positive) and find K of M positive donors, the posterior distribution of positive donors was modeled as: p+ = Beta (K + 1; M − K + 1). Finally, only municipalities with at least 20 BDs analyzed were considered in the logistic regression. Intercooled Stata 7.0 software (Stata Corporation, College Station, TX, USA) was used for statistical data analysis. Significance was established at *p* < 0.05. A map showing the TOSV-positive BDs and the sand fly abundance for each municipality was created in the open source Geographic Information System (GIS) software QGIS 2.18 (www.qgis.org).

## 3. Results

### 3.1. TOSV Serological Studies

As shown in Table 2, 151 ELISA-reactive samples were found in Bologna and only two samples in Ferrara.

The ELISA-reactive samples were then tested by IIF and MN (Table 3). None of the BD sera collected from the Ferrara province tested positive by IIF or MN. On the contrary, among the 151 ELISA reactive sera coming from Bologna province, 59 showed specific antibody by IIF and 61 by MN, with neutralizing antibody titers ranging from 1/40 to 1/160. IIF revealed also five reactive samples for anti-SFSV IgG, 23 for anti-SFNV IgG, and three for anti-SFCV IgG.

Eighteen out of 151 ELISA-reactive samples exhibited discordant results when tested by IIF and MN; 10 specimens tested positive by MN and negative by IIF, while 8 samples tested MN-negative and IIF-positive (Table 4). A complete agreement between the two second-level tests was obtained in 51 positive samples and 82 negative samples. None of the 23 samples that tested positive for anti-SFSV IgG, anti-SFNV IgG, or anti-SFCV IgG by IIF exhibited anti-SFSV neutralizing antibodies.

**Figure 2:**
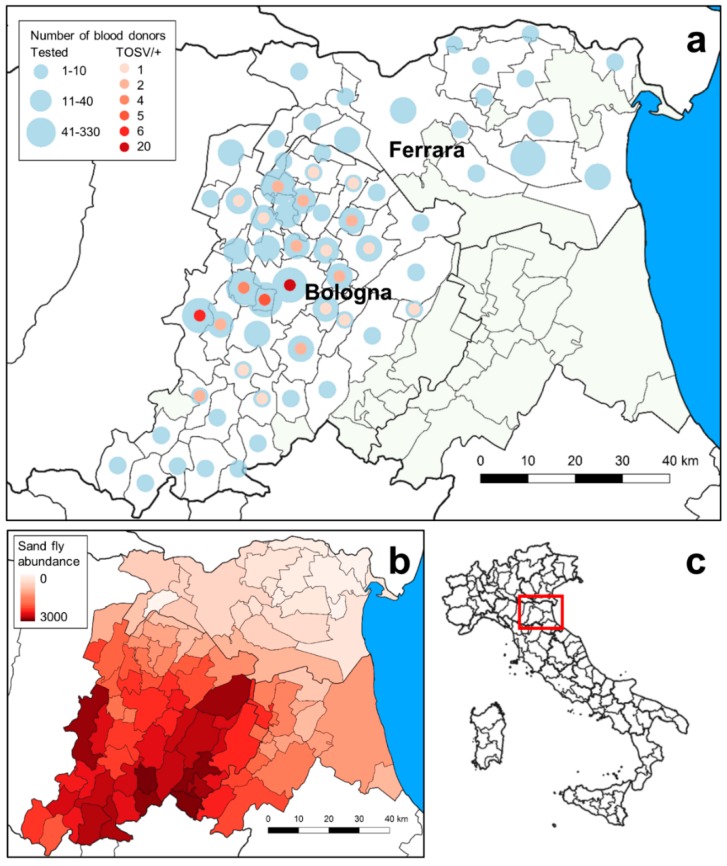
(**a**) Distribution of blood donors included in the study (light blue circles) and anti-TOSV IgG-positive individuals (red-colored circles) for each municipality in the surveyed area (circle areas are proportional to the effective number of individuals, reference number in the legend). (**b**) Choropleth map of the estimation of sand fly abundance obtained by the model for each municipality (from 0 to 3000 sand flies sampled). (**c**) Location of the surveyed area in Northeastern Italy.

Serum samples were defined as positive for anti-TOSV IgG when reactive by ELISA and by at least one second level test (IIF and/or MN, Figure 1). Sixty-nine BDs tested positive for anti-TOSV antibodies in the Bologna province (51 MN-positive/IIF-positive, 10 MN-positive/IIF-negative, and eight IIF-positive/MN-negative) and none in the Ferrara province, respectively. Thus, seroprevalence for TOSV was 6.8% in the Bologna province and 0% in the Ferrara province. The highest number of anti-TOSV positive samples was from BDs residing in the municipalities of Bologna, Casalecchio di Reno, Zola Predosa, and Valsamoggia (Figure 2a and Supplementary Materials).

### 3.2. Sand Fly Abundance Modelling

The implemented model produced an estimation of total sand fly number at 250 m × 250 m resolution (see Supplementary Materials). The observed seroprevalence was strongly associated with the estimated sand fly abundance (Figure 2b), as pointed out by the results of the binomial logistic regression model (*p* < 0.001). According to this model, 75.5% of the variability observed in the prevalence of TOSV-IgG positive BD per municipality was explained by the estimated abundance of sand flies in the site following the equation: Logistic (p+) = 0.29 × log10 (SA) − 2.651 (R^2^ = 0.755). The equation indicates that the prevalence of TOSV antibody in BDs (logistic transformation) is positively associated to the abundance of the vectors in the different municipalities.

## 4. Discussion

In this study, we observed that anti-TOSV positive BDs resided mainly in municipalities of the Bologna province, while no active TOSV circulation was observed in the Ferrara province; these findings match with estimated abundance of sand flies in these two provinces of Northeastern Italy. Between 2010 and 2013, 143 cases of neuroinvasive infection caused by TOSV were identified in the Emilia-Romagna region, Northeastern Italy; the highest number of TOSV cases was recorded in the Bologna province and the lowest number in the Ferrara province [17,18]. In line with the distribution of symptomatic TOSV cases, our serosurvey identified a high circulation of TOSV in the Bologna province, with a seroprevalence of 6.8%, while no TOSV circulation was observed in the Ferrara province.

The seroprevalence of TOSV was previously investigated in other Italian regions; studies on the antibody prevalence rate of TOSV were performed by employing ELISA in Piedmont (Northern Italy), Tuscany (Central Italy), and Sicily (Southern Italy), highlighting seroprevalence rates ranging from 3% to 33% with a north to south gradient [8,22]. Other studies from Southern Europe indicate seroprevalence rates for TOSV ranging from 12% to 38% by employing either ELISA or IIF [14,23,24,25,26]. Considering only the results obtained by ELISA, the abovementioned seroprevalence rates would be comparable to the data obtained in this study; 13% of our BD samples were reactive by ELISA. Nevertheless, we decided to define a serum sample as positive for anti-TOSV IgG when reactive by ELISA and by at least one second level test in order to increase the specificity of our results. Thus, the employment of a stringent condition for seropositivity in the current study can explain the lower TOSV seroprevalence rate in Northeastern Italy as compared to other Italian and Southern European regions, despite the high incidence of TOSV neuroinvasive cases in this area.

To our knowledge, few studies assessed TOSV seroprevalence in Europe by a combination of tests [27,28]. In addition, only one study employed MN as a confirmatory test, showing a 5% seroprevalence for TOSV in Turkey [27], which is in line with our results. In agreement with our findings, a study performed in 2011 on 2625 BD plasma samples collected in Southeastern France showed ELISA and MN rates of 7.3% and 2.1%, respectively [29].

In the current study, 18 out of 151 ELISA-reactive samples showed discordant results when tested by IIF and MN. Ergunay and colleagues previously observed this phenomenon in TOSV infection [30], and other studies involving arboviruses such as yellow fever virus also showed that the correlation of ratio/titers for IIF to MN was low [31]. Phlebovirus neutralization is a complex phenomenon involving several antigens on viral glycoproteins that may display considerable variation [29]. Further, neutralizing epitopes can depend on protein tertiary structures that may be lost during IIF assay development [30,32,33].

We observed that 23 samples tested positive for anti-SFSV IgG or anti-SFCV IgG by IIF, but all of them were negative by SFSV MN, which is the optimal test in terms of specificity. As the serological cross-reactivity among phleboviruses for certain serological tests is a known phenomenon [34], this result could be explained with the presence of phleboviruses other than TOSV and SFSV circulating in Northeastern Italy and infecting BDs. Consistent with this hypothesis, several phleboviruses with unknown pathogenicity were detected in sand flies in the Bologna province [35,36].

The relation between an arthropod-borne pathogen and the abundance of its vector is difficult to demonstrate without detailed entomological data. We overcame the unavailability of sand fly data at the municipality level implementing a model to obtain a point estimate of the sand fly abundance. For this estimation, we used some physical variable (land use and Digital Terrain Model), the Normalized Difference Vegetation Index (June–September 2014), and weather data referred to 2001 to 2012 period, which is long enough to represent the year of sampling. This approach allowed to show that abundance of vectors parallels the rate of TOSV-positive donors in each municipality, identifying the abundance of sand flies as a factor that strongly influences TOSV seroprevalence in humans. The low abundance of sand flies in the Ferrara province, as highlighted by vector analysis, is in line with the lack of anti-TOSV IgG in BDs living in this area. A high abundance of sand flies was estimated in some municipalities of the Bologna province, which coincides with areas that exhibit the highest TOSV seroprevalence.

The main limitation of this study is that the study group exclusively comprised healthy adults, thus the results cannot be generalized to the overall population of the examined provinces.

## 5. Conclusions

The serosurvey carried out in 2014 showed different circulation of TOSV in different provinces of Northeastern Italy, with a 6.8% TOSV seroprevalence in the Bologna province. Information provided by this study may be useful for public health authorities, as our findings raise concerns about the risk of TOSV transmission to virus-naive individuals by blood transfusion or organ transplantation; TOSV has been isolated from human blood on several occasions [37], and this suggests that a potential risk for virus transmission by iatrogenic means exists.

## Figures and Tables

**Table 1:** Entomological data employed to implement the model with reference to the provinces where sand fly collection took place (July–August 2014).

Entomological Data	Bologna Province	Ferrara Province	Ravenna Province
Number of traps	32	21	9
Traps collecting sand flies	25	2	2
Collected sand flies	1688	2	30
Maximum number of sand flies per trap	1081	1	27

**Table 2:** Detection of anti-TOSV IgG by ELISA in blood donors from the Bologna and Ferrara provinces (Northeastern Italy), April–July 2014.

Detection of Anti-TOSV IgG by ELISA	Bologna Province (Total BDs = 1008)	Ferrara Province (Total BDs = 200)
Number of positive BD samples for anti-TOSV IgG	123	2
Number of border line BD samples for anti-TOSV IgG	28	0
Total ELISA-reactive samples	151 (15%)	2 (1%)

BD, blood donor.

**Table 3:** Detection of anti-TOSV IgG by indirect immunofluorescence assay (IIF) and microneutralization test (MN) in BDs that previously tested positive or borderline by ELISA-IgG. IIF: indirect immunofluorescence assay; MN: microneutralization assay; TCID_50_: 50% Tissue Culture Infective Dose.

Test to Detect Anti-TOSV IgG	Bologna Province (*n* = 151)	Ferrara Province (*n* = 2)
IIF	59	0
MN	61	0
TOSV MN (100TCID_50_) titers	1/40 (*n* = 22) 1/80 (*n* = 27) 1/160 (*n* = 12)	

**Table 4:** Results obtained by indirect immunofluorescence assay (IIF) and microneutralization test (MN) in sera from 151 BDs that tested positive or borderline by ELISA.

Second Level Tests	MN+	MN−	Total
**IIF+**	51	8	59
**IIF−**	10	82	92
**Total**	61	90	151

## Supplementary Materials

The following are available online at https://www.mdpi.com/2076-2607/8/2/145/s1.
