# Supplementary material for: Detection of Specific Antibodies against Toscana Virus among Blood Donors in Northeastern Italy and Correlation with Sand Fly Abundance in 2014
Source: Microorganisms. 2020 Jan 21;8(2):145. doi: 10.3390/microorganisms8020145 (PMC7074719; doi:10.3390/microorganisms8020145)
Supplement: Supplementary file 1 [file microorganisms-08-00145-s001.pdf]

Supplementary material

Materials and methods

Modeling of the sand fly abundance

We used a Generalized Linear Model (GLM) to estimate the sand fly abundance using, as dependent variable in a Poisson regression, the total number of sand flies per trap over the period July 3-August 21, 2014, and evaluating the factors that could be associated with the total number of sand flies. Remote sensing variables used for modeling abundance of sand flies in 2014 in the provinces of Ferrara, Bologna and Ravenna were the following;

1) Mean Normalized Difference Vegetation Index (NDVI) at 250m resolution calculated from June to September 2014 (MODIS images - ARPAE);

2) 9 Bioclimatic Indexes extracted from EURO LST time series at 250 m resolution 2001-2012 (Metz et al. 2014). All these indexes were referred to temperatures and were, following the "Bioclim" definition of Hutchinson et al. (2009), included; BIO1: Annual mean temperature (°C*10); BIO2: Mean diurnal range (Mean monthly (max – min tem)); BIO3: Isothermality ((bio2/bio7)*100); BIO4: Temperature seasonality (standard deviation * 100); BIO5: Maximum temperature of the warmest month (°C*10); BIO6: Minimum temperature of the coldest month (°C*10) ; BIO7: Temperature annual range (bio5 – bio6) (°C*10); BIO10: Mean temperature of the warmest quarter (°C*10); BIO11: Mean temperature of the coldest quarter (°C*10).

3) Corine Land Cover 2012 from Copernicus Images;

4) DTM extracted from SRTM Images.

All images were resampled at 250 m resolution and cropped inside the study area using QGIS 2.18. Abundance model were carried out in R version 3.3.2 (R Development Core Team 2005) using the lme4, lattice, Tweedie, boot, raster, sp and rgdal packages. A GLM with log link and poisson error was used for modelling sand fly abundance from the beginning of July to the end of August 2014 (specimens sum) in 62 CO_2 traps correlated to the 12 explanatory variables. A 6-fold Cross Validation was used to validate the model and the deviance explained to evaluate accuracy. This means that data set was split into 6 equal parts, the model was applied to all data except one part, data of excluded part were used as control; the procedure was applied for each part of the data.

Correlation of human TOSV-seroprevalence and sand fly abundance

A binomial logistic regression model was used to evaluate the correlation between the TOSV seroprevalence at municipality level and the sand fly abundance (SA), estimated as average of sand flies per trap per municipality according to the obtained model. To reduce the high variability typical of the vector population, the abundance was log10 transformed before binominal logistic regression model analysis. For each municipality, results from the prevalence observed in blood donors were analyzed separately by means of binomial logistic regression with log10 (SA) as covariate. Intercooled Stata 7.0 software (Stata Corporation, College Station, TX, USA) was used for statistical data analysis. Significance was established at $p < 0.05$. The binomial logistic regression model was highly significant ($p<0.001$).

Results

Medialization of the sand fly abundance

The first four variables ranked by the model are reported in Table S1. A 92% value was obtained by the 6-fold Cross Validation, and the cumulative deviance explained by the first 4 variables to evaluate accuracy is

reported in Table S2. The output of the model (estimated number of sand flies per trap) is graphically represented in Figure S1.

Table S1. First four variables ranked by the model

Variable	ESTIMATE	STD.ERR	t-VALUE	p
(Inter.)	63,488	3,569	17,788	<0.001
BIO2	0,808	0,046	17,636	<0.001
BIO3	-4,269	0,136	-31,384	<0.001
BIO4	-0,012	0	-41,424	<0.001
BIO7	0,222	0,012	18,731	<0.001

Table S2. Cumulate deviance explained (% DEV) by the first four variables of the model

RANK	VARIABLE	% DEV	AIC
1	BIO2	28,98	6.266
2	BIO3	40,00	5.315
3	BIO4	78,62	1.978
4	BIO7	82,54	1.641

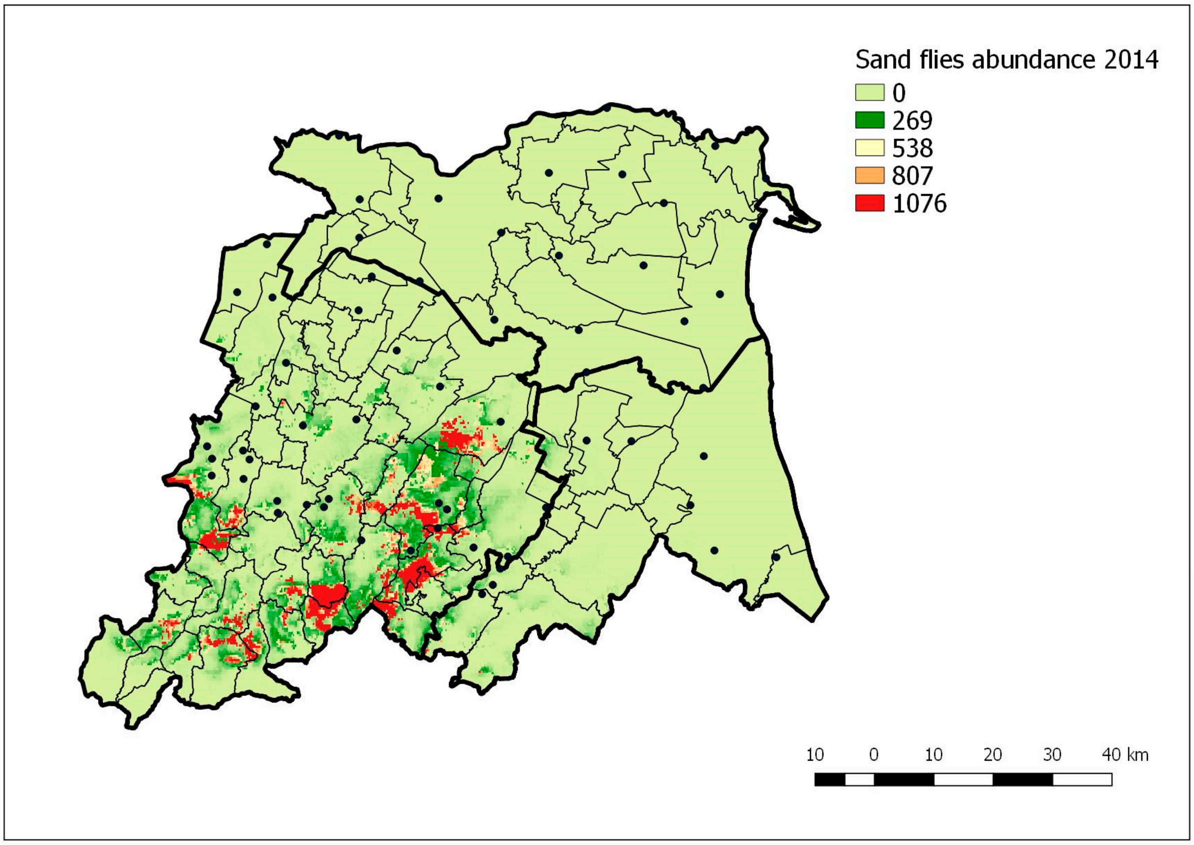

Figure S1. Graphic representation of estimated number of sand flies per trap with reference of location of the traps (black dots).

Correlation of human TOSV-seroprevalence and sand fly abundance

A binomial logistic regression model was used to evaluate the correlation between the TOSV seroprevalence at municipality level and the sand fly abundance (SA) (1).

$$\text{Logistic}(p+) = \text{Ln}[p+/(1-p+)] = a + b \log10(SA) \quad (1)$$

To avoid 0 prevalence result and to consider the uncertainty introduced by sampling on the true prevalence estimation the mean of a beta function was used. The beta transformation was applied to the prevalence observed in each municipality. Therefore, if 0 positive donors were observed out of 27 donors analyzed (Poggio Renatico municipality) the real prevalence (p+) estimated was 0.04 and the logistic transformation of (p+) was -3.3. Using Bayes' theorem where no prior knowledge of the prevalence (P) in blood donor population is available, the fraction of positive donor could be assumed to follow a Beta distribution (Vose 2006). If we assume a uniform [0.1] prior distribution for P+ (the probability of being positive) and find that K of M positive donors, the posterior distribution of donors was modeled as reported in (2).

$$P+ = \text{Beta}(K + 1; M - K + 1) \quad (2)$$

Finally, the logistic regression included only municipalities where at least 20 donors were examined for TOSV IgG; data are summarized in Table S3.

Table S3. Relative prevalence for TOSV IgG and sand fly abundance estimated by the model for each municipality, Bologna and Ferrara provinces, northeastern Italy.

Province	Municipality	Tested BD	TOSV-positive BD	TOSV relative seropre-valence	SF Average	Minimum N of SF*	Maximum N of SF*
Bologna	Bologna	330	20	6.1	33	0	1,608
	Casalecchio di Reno	46	5	10.9	4	0	29
	Castel Maggiore	25	2	8.0	13	0	121
	Castello d'Argile	41	2	4.9	1	0	27
	Castenaso	26	2	7.7	66	2	2,046
	Granarolo dell'Emilia	25	1	4.0	32	0	408
	Monte San Pietro	23	2	8.7	171	0	4,382
	Pianoro	23	2	8.7	161	0	7,846
	San Giovanni in Persiceto	30	1	3.3	8	0	524
	Valsamoggia	65	6	9.2	699	0	39,388
	Zola Predosa	55	4	7.3	5	0	32
Ferrara	Comacchio	31	0	0.0	0	0	0
	Ferrara	31	0	0.0	0	0	1
	Fiscaglia	24	0	0.0	0	0	0
	Ostellato	60	0	0.0	0	0	0
	Poggio Renatico	27	0	0.0	0	0	0

SF; sand flies, N; number, * Value estimated by the model within the related municipality;

The 75% of the variability observed in the prevalence of positive BD per municipality is explained by the abundance of sand flies, expressed as log (10) following the equation (3).

$$\text{Logistic}(P+) = 0.29 \log10(SA) - 2.651 \quad (3)$$

The coefficient 0.29 has a 95 coefficient interval (CI) of 0.208-0.395 and the intercept has a 95 CI of-2.791-2.410. The Figure S2 represents the logistic transformation of the prevalence (p+) of BD in each municipality (y axis) against the log(10) of sand fly mean abundance (x axis). The equation indicates that the prevalence of TOSV in BD (logistic transformation) is positively associated to the abundance of the vectors in the different municipalities.

Figure S2. Binomial logistic regression of TOSV prevalence (P) in BD and sand fly abundance index (log10SA).

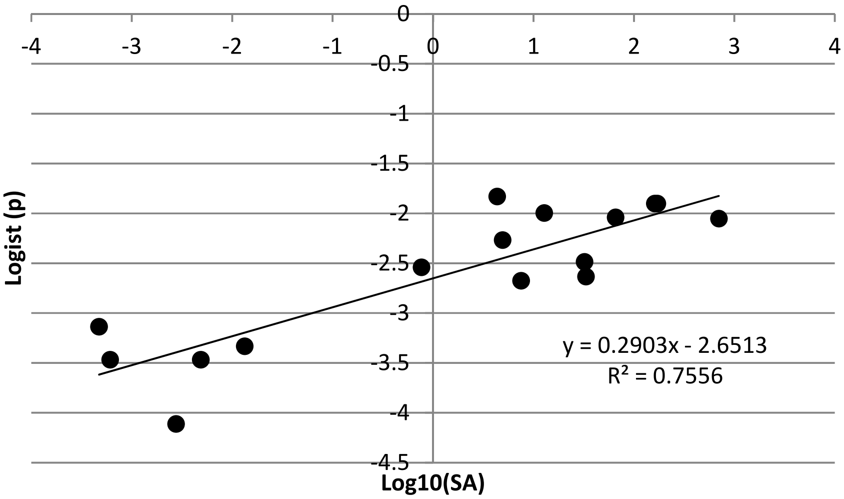

References

Hutchinson M., Xu T., Houlder D., Nix H., McMahon J. (2009). ANUCLIM 6.0 User's Guide. Australian National University, Fenner School of Environment and Society, Canberra.

Metz M., Rocchini D., Neteler M. (2014). Surface temperatures at the continental scale: Tracking changes with remote sensing at unprecedented detail. Remote Sensing. 2014, 6(5): 3822-3840.

Vose D (1996). Quantitative Risk Analysis: a guide to Monte Carlo simulation modelling. New York: Wiley.
